# Textile Supplies for Hospitals

**Published:** 1916-03-18

**Authors:** 


### TEXTILE SUPPLIES FOR HOSPITALS.

### VII. Wool Clothing.

Time and change have produced numerous substitutes for the true flannel which is the best approved fabric for ward wear, and which was once worn nearly universally. Of these alternative materials it cannot be said that any one presents an improvement at all points. Some have proved themselves more taking in appearance, like the fancy flannels and shirtings sold in striking colours and designs. Most of them are materially lower in price, and notably so in the case of what is now called "flannelette," and in its earlier years was less ambiguously known as "Wellington shirting." The immense popularity of the close-fitting knitted garments that are bought ready-made completes the list of causes that have deposed flannel from its old place in the general market. The visible falling-off in general favour is by no means to be ascribed to defects inherent in the article itself.

### What is Flannel?

"Flannel" is a name somewhat loosely applied to warm and woolly fabrics in general, but it is susceptible of close definition, and the Merchandise Marks Act has repeatedly been used against those who have sold as being flannel cloth not all-wool. "Flannel," as a designation standing by itself, implies a warranty that the goods are all-wool, but the qualifications of it, like "Ceylon flannel," "Bavarian," or "Canton" flannel, are notices simply that the material is such as is usually sold under those names, and their implication is quite separate.

The true flannels are sold under qualifying names indicative of their quality, and faintly misleading as to their origin. "Yorkshire" flannels, for example, are made not in Yorkshire but at Rochdale, Lancashire, the old centre in which the flannels woven in the Yorkshire hills were marketed a century or so ago. "Lancashire" flannels, differing somewhat in quality, are also Rochdale-made, and the lineal successors of the old indigenous type. "Flannel" is a word itself of Welsh derivation, and "Welsh" flannels are made in the Principality and closely imitated in Lancashire. "Saxony," a name given to soft superfine flannels, signifies no more than that they are made of what is variously known as merino, Botany, or Saxony qualities of wool. "Shetland," which in the stricter sense implies either island origin or manufacture from Shetland wool, is used sometimes to describe flannels which have been napped or raised lightly on the back and face.

### Some Properties of Flannel.

Flannel is technically a near relation of the blanket, lighter in weight and finer in texture, but made from similar sorts of wool and intended for warmth more than for decoration. White flannel is made upon a large scale, although it is less used for hospital work than the "natural" mixture shade produced by blending together white and coloured wool. Flannel whitened by sulphur stoving in course of the finishing processes tends to yellow with washing and age, and the tone is a less pleasing one than the brown deliberately obtained by blending. White flannel is blued with laundry blue to enhance its whiteness for use as bandages, and although in any case wool soils less rapidly than cotton, the disposition to use coloured flannel for bed-jackets needs no explanation. The "natural" shade soils still less quickly than white, while red flannel has a cheerfulness that is not without therapeutic value.

Like other woollens, flannel has a natural propensity to shrink, and alkalies, warmth, moisture, and friction assist the shrinkage. These conditions are provided in the normal laundry machine, and it is commonly found worth while to wash flannels by hand, removing the last trace of soap from them carefully. The tendency to shrink varies with the quality of flannel, and is greatest ordinarily in the finest and softest kinds, manufactured from the more wavy and closely serrated wools. The conditions are best met by a compromise upon a medium quality, neither too harsh to the skin nor too prone to shrink. No wool is naturally unshrinkable, although some fancy flannel cloths and many knitted garments are so described. The materials sold under the name are put through a chemical process, usually one of chlorination, whereby the minute scales of the wool fibre are removed. The character of the wool is modified and its durability is at least not enhanced by the treatment, and at all events, for the making of loose-fitting garments, adaptable to all comers, an untreated fabric of moderate shrinking propensities is to be preferred.

Flannel is chosen for warmth, and nothing is superior to wool in that respect. Mixed flannels containing cotton, although cheaper in the first instance, are less bulky and less efficient non-conductors from the beginning. The disparity in warming power increases with age, for in wear the wool component leaves the fabric first, the cotton remaining behind. The rival flannelette, when its pile has been scrubbed away, remains a mere calico, while the very shrinking-in of the true flannel tends to restore its original virtue throughout its life.

### Staff Clothing.

The distinction between ward and staff clothing is great, but both sets of parties are wearers of wool, and the matter of outdoor uniforms has an undeniable importance. Nurses' cloaks come into store in the made-up state, and their character is

Note.—Previous articles in this series appeared on December 18, 1915, and January 8 and 22, February 5 and 19, and March 4, 1916. The next article will treat of "Sheets and Cottons."

only in part a question of the material from which they are cut and sewn. Fabrics suitable for their purpose are chosen by the cloak-maker, and his staple material is serge. In an eminent degree a good serge combines the elements of lightness and warmth, coupled with trimness and durability.

In examining a standard serge from which great numbers of nursing cloaks are made it is found to have a distinctly crisp touch. The not disagreeable hardness comes in the first instance from the cross-bred wool of which the stuff is made, a wool that is rather more repellent of water than fine, spongy merino. The warp threads, which have to bear the greater strain, are twofold worsted, and the weft is single-worsted yarn. The cloth is springy, and smartly recovers from creasing. Its surface shows a rather fine twill, due to weaving two and two threads alternately, and the weave is chosen designedly to prevent the slipping of threads one over the other, which causes some hard serges to give way near the seams. The face has a light cover of fibre, produced not by scratching or raising the pile, but by milling or hammering the fabric in course of finishing. This face of fibre, besides covering the interstices and improving the warmth, serves to delay the shininess which comes more or less to all worsted fabrics after a certain length of wear. The weight is a summer one of 9½ ounces per yard, 54 inches wide, and for winter a similar but heavier and darker cloth of 15-ounce weight is substituted. The colour is blue, and as fastness of colour is a desideratum the effective choice is between indigo and alizarin blue. The latter is hardly inferior to the former in resistance to light and weather, and possesses the advantage of being fast against rubbing, whereas indigo tends to soil any white garment over which it is worn.

### Standard Serges.

The serges in point have been rainproofed by a patent process, in course of which they are first impregnated with a chemical which is itself highly soluble, the acetate of alumina. As a second measure of protection the cloth is treated with an imperceptibly fine smear of wax, distributed equally all over it. The effect is greatly to increase the natural repellency without materially altering the appearance or character of the cloth. It may be worth adding that the water-resisting power of cloaks treated according to this system is rather increased than diminished if the wet garment is dried before the fire.

The worsted serge that has been described is, on the whole, rather smarter in appearance than the woollen serge that may be supplied in its place. The woollen serge more closely resembles the serge that is served out to the Navy, and it is made with worsted in the warp direction with a woollen thread for weft. Either kind can be dyed in a choice of colours, none probably more serviceable than blue. Brown, claret, and green are less dependable than blue in ordinary times, and are particularly likely to prove unsatisfactory in days when the supply of colouring matters is short. Next to blue, one of the most widely used colourings is mixture grey in worsted serges, a colour that is obtained by dyeing combed wool and blending together light and dark shades before proceeding with the manufacture.

For the hottest days of summer nothing is cooler for outdoor than a cloak of mohair. The material has more resiliency than serge, and its lustre lends it a distinction. It is light in weight and comparatively open in texture, weighing perhaps one-fourth less than the regular summer serge. The material is the reverse of clinging; it has no tendency to accumulate dust, and is thus peculiarly fitted for summer wear.

The general practice of the hospitals in preferring such goods as have been described squares with the lessons of experience in general, and it could be modified only in non-essentials. There may be ways in which the same basic materials could be worked up to similar advantage, but probably not to greater advantage in any event. Where the materials are well chosen and manufactured straightforwardly towards the production of a simple cloth there is not much to choose between alternative means, or anything considerable to gain by departure from the accepted course.

### Cheap Woollens.

Cheaper fabrics are made than those which can be produced from all-wool, and it may be conceded that surprisingly good wear is returned by some of them. The word " shoddy " need not be applied in its most opprobrious sense to cloths that are certainly made from second- and third-hand materials. Cloaks can be made from the Melton cloths that are reconstituted from soft and short rag wool woven across a cotton warp and from cotton-warp serges made from the remains of harder and more cottony rags. The articles are made less for hospitals than for individuals wanting uniforms at the lowest first cost, but if the value for money is remarkable it is more advantageous in all stages to buy the better goods, and none the less in a time when prices are higher all round. The matter is one of comfort and appearance, as well as of warmth and wear. The cheaper cloth manufactured from material of dark colour, which has been dyed at least once before, does not take up the latest of the dyes uniformly. In the nature of the case these inferior cloths are relatively heavier than their betters, and they grow shabby much earlier. No more is to be got out of any textile fabric than is put into it, and a basis of rags and cotton is in any event less promising than one of unimpaired wool. The clamour against rags raised because in their previous incarnation they may have been dirty misses the point. Their dirt is fairly effectually removed in the course of their re-manufacture, but the wool is not rehabilitated. The material is progressively impoverished by the incidents of its wear, its disintegration into loose fibre again, and its re-manufacture into cloth; and no amount of explanation serves to remove the fact that in course of its progress from wool to cloth, and cloth to rags, and rags to cloth again, woollen material grows continually less to be desired.

